# Management of Hereditary Spastic Paraplegia: A Systematic Review of the Literature

**DOI:** 10.3389/fneur.2019.00003

**Published:** 2019-01-22

**Authors:** Marta Bellofatto, Giovanna De Michele, Aniello Iovino, Alessandro Filla, Filippo M. Santorelli

**Affiliations:** ^1^Department of Neurosciences and Reproductive and Odontostomatological Sciences, Federico II University, Naples, Italy; ^2^Molecular Medicine, IRCCS Fondazione Stella Maris, Pisa, Italy

**Keywords:** spastic paraplegia, genetic, therapy, literature review, quality of study

## Abstract

The term hereditary spastic paraplegia (HSP) embraces a clinically and genetically heterogeneous group of neurodegenerative diseases characterized by progressive spasticity and weakness of the lower limbs. There currently exist no specific therapies for HSP, and treatment is exclusively symptomatic, aimed at reducing muscle spasticity, and improving strength and gait. The authors set out to perform a comprehensive systematic review of the available scientific literature on the treatment of HSP, applying Cochrane Collaboration methods. The Google Scholar, PubMed and Scopus electronic databases were searched to find relevant randomized control trials (RCTs) and open-label interventional studies, prospective, and retrospective observational studies of supplements, medications, and physical therapy, as well as case reports and case series. Two authors independently analyzed 27 articles selected on the basis of a series of inclusion criteria. Applying a best-evidence synthesis approach, they evaluated these articles for methodological quality. A standardized scoring system was used to obtain interrater assessments. Disagreements were resolved by discussion. The 27 articles focused on pharmacological treatment (*n* = 17 articles), physical therapy (*n* = 5), surgical treatment (*n* = 5). The drugs used in the 17 articles on pharmacological therapy were: gabapentin, progabide, dalfampridine, botulinum toxin, L-Dopa, cholesterol-lowering drugs, betaine, and folinic acid. Gabapentin, progabide, dalfampridine, and botulinum toxin were used as antispastic agents; the study evaluating gabapentin efficacy was well-designed, but failed to demonstrate any significant improvement. L-Dopa, cholesterol-lowering drugs, betaine, and folinic acid were only used in specific HSP subtypes. Two of the three studies evaluating cholesterol-lowering drugs (in SPG5 patients) were well-designed and showed a significant reduction of specific serum biomarkers (oxysterols), but clinical outcomes were not evaluated. The articles focusing on physical treatment and surgical therapy were found to be of low/medium quality and, accordingly, failed to clarify the role of these approaches in HSP. Despite recent advances in understanding of the pathogenesis of HSP and the possibility, in several centers, of obtaining more precise and rapid molecular diagnoses, there is still no adequate evidence base for recommending the various published therapies. Well-designed RCTs are needed to evaluate the efficacy of both symptomatic and pathogenetic treatments.

## Introduction

Hereditary spastic paraplegia (HSP) refers to a clinically and genetically heterogeneous group of neurodegenerative diseases characterized by progressive spasticity and weakness of the lower limbs. This lower limb impairment is caused by relatively selective distal axonal degeneration that involves the longest axons of the corticospinal tracts. On clinical grounds, HSP are highly heterogeneous with both pure and complex forms being possible. Onset can occur at any age and congenital, pediatric, and adult-onset manifestations are seen worldwide (1). The modern genetic classification of the different forms of HSP is based on mode of inheritance, chromosomal locus, and causative mutation (if known). HSP can show an autosomal dominant (AD), autosomal recessive (AR), X-linked or maternal (mitochondrial) pattern of inheritance. To date, 85 different spastic gait disease loci have been identified in 79 known causative genes (1). The overall prevalence of HSP has been estimated to be 1.8/10^5^ for both AD and AR forms (2) with age at onset and disease progression also varying greatly within the different forms (3). There currently exist no specific therapies able to prevent, delay, or reverse the progressive disability in HSP. Treatment is exclusively symptomatic and aimed mainly at reducing muscle spasticity and urinary urgency, and improving strength and gait. Therapeutic options include physical therapy, oral antispastic drugs (baclofen, progabide, dalfampridine), botulinum toxin therapy, and surgical baclofen pump implantation. However, clinicians currently have no evidence-based guidelines or recommendations to help them select the most suitable treatment, and to date no critical overview of the therapeutic *armamentarium* has been provided. The present systematic review of current evidence on treatment in HSP was conducted with the aim of formulating possible management guidelines and recommendations on drug therapy for these conditions.

## Materials and Methods

Three large datasets—PubMed, Scopus (both last accessed in April 2018) and Google Scholar (May 2018)—were searched using the terms “hereditary spastic paraplegia treatment” OR “hereditary spastic paraplegia drugs” OR “hereditary spastic parapalegia rehabilitation” AND/OR “rehabilitation therapy” and “hereditary spastic paraparesis.” The search was supplemented by citation tracking of researchers publishing in pertinent areas, and the reference lists of relevant clinical reviews and abstracts published in conference proceedings were also consulted.

After initial screening of the results and elimination of duplicates, the remaining articles underwent further screening. To be selected for inclusion in the study, papers had to meet a series of criteria: they had to be full-text articles written in English that focused specifically on populations affected by HSP submitted to therapeutic interventions. In addition, preference was initially given to randomized controlled trials (RCTs) that reported data on therapy for children and adults with HSP, but this criterion was subsequently relaxed to allow the inclusion of other types of study relevant to HSP therapy. At this stage of the selection process, three reviewers independently screened the titles and abstracts of articles identified by the initial search to evaluate their compliance with the inclusion criteria. In all cases, however, it proved necessary to read and evaluate the full article in order to determine its suitability for inclusion.

## Data Analysis

The design and quality of the included studies were evaluated. Their methodological quality was assessed using the total score recorded on an 11-item scoring list (Appendix 1) (4), where each item was scored as 0 (“no”), 1 (“not sure”), or 2 (“yes”). The maximum total score of 22 was taken to indicate excellent quality. This aspect was evaluated independently by two authors who, by consensus, agreed to score the quality of papers as low if their mean total was ≤ 8, moderate if it was between 9 and 16, and good if it was ≥17. If necessary, any disagreement in scoring between the two assessors was resolved by discussion with a third assessor until consensus was achieved.

## Results

**Figure 1:**
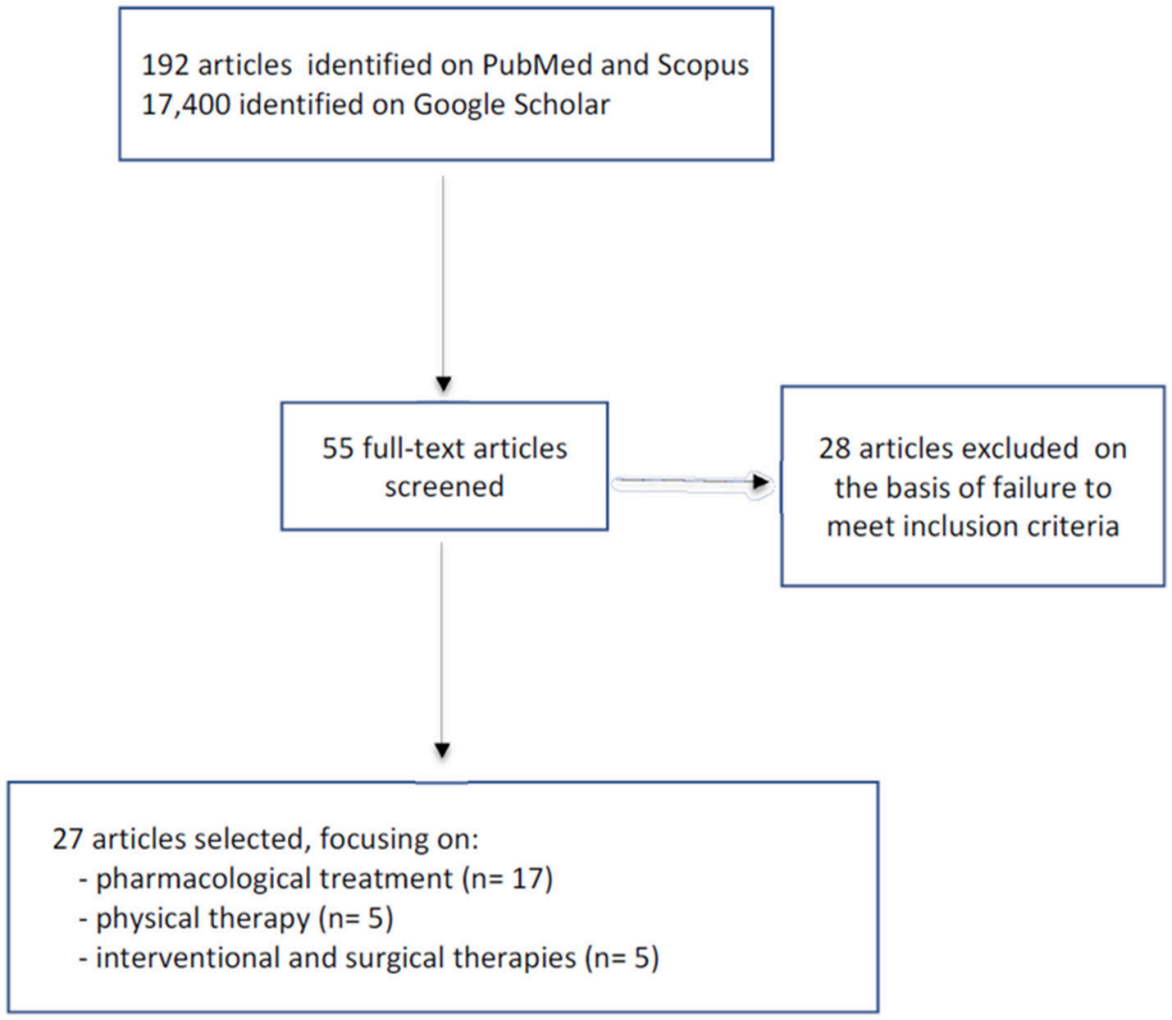
The search strategy.

The PubMed and Scopus search yielded a total of 192 articles, while 17,400 were found in Google Scholar. The titles and abstracts were screened and any duplicates were eliminated. At this point the remaining 55 articles underwent further screening; of these, 28 were excluded because they failed to meet the inclusion criteria (not dealing specifically with HSP). The final selection comprised 27 articles: 17 dealing with pharmacological treatments, five with physical therapy, and the last manuscripts discussing interventional and surgical therapy (Figure 1).

### Pharmacological Treatments

The selected papers dealing with pharmacological treatments investigated the use of the following drugs.

#### Gabapentin

Gabapentin is an antiepileptic and antinociceptive drug whose prevalent mechanism of action is inhibition of calcium currents via high-voltage-activated channels containing the α2d-1 subunit, leading to reduced neurotransmitter release and attenuation of postsynaptic excitability (5). To evaluate its antispastic action, gabapentin was tested in a double-blind, prospective, crossover placebo-controlled trial in patients with SPG4-linked hereditary spastic paraplegia (6). Ten adult patients were included and were randomly allocated to two treatment regimens: the first received gabapentin for a period of 2 months, which was followed by a washout period of 10 days, a drug-free interval of 1 month, and finally placebo therapy for 2 months; the second group received the same treatments in the opposite order. The gabapentin treatment consisted of a starting dose of 2,400 mg/day, titrated up over 10 days to a maximum dose of 4,000 mg/day. Outcome was rated according to objective clinical measures—the authors used a combination of the MS Impairment Scale (7) and the SPATAX (8) diagnostic form—and self-reported measures, as well as paired transcranial magnetic stimulation parameters for evaluating motor cortical excitability. Objective clinical examinations and self-reported parameters were assessed at baseline, at the end of the treatment period, and at the end of the placebo treatment period. Overall, no statistically significant clinical differences (including intra-cortical excitability) were found between gabapentin and placebo. The quality of Sills' study was rated as good on the basis of the score of 19/22 assigned by the assessors, both of whom, however, urged caution, given the very small size of the sample considered.

#### Progabide

Progabide is a highly selective GABA receptor agonist. In a double-blind crossover trial involving two 14-day treatment periods, progabide was compared with placebo in 16 adults with spasticity, two of whom had a clinical diagnosis of HSP (with an unspecified molecular etiology) (9). Progabide was administered at a median daily dosage of 24.3 mg/kg (range 14.3–32.7 mg/kg). Outcome measures were: spastic hypertonia (evaluated, on a semi-quantitative scale, by measurement of the angle at which the stretch reflex appeared in response to mobilization of the limb at gravity speed) and reflex responses and flexor spasms (both evaluated using semi-quantitative scales). Progabide was found to significantly reduce spastic hypertonia (*p* < 0.01), reflex responses (*p* < 0.01), and flexor spasms (*p* < 0.05).

The quality of this study was rated as moderate (16/22). However, only two patients had an HSP-related form of spasticity. Furthermore, the treatment was short-term and the selected outcomes were not clinically significant.

#### Dalfampridine (4-Aminopyridine)

Dalfampridine is a voltage-dependent potassium channel blocker used to improve walking ability in patients with multiple sclerosis. We reviewed two prospective, uncontrolled, open trials in which dalfampridine, 10 mg, was administered orally twice daily for 2 weeks. The first study included 12 adult patients affected by HSP who were treated for 15 days (10). Efficacy was assessed on the basis of walking ability improvement, evaluated using the Timed-25-Foot Walk Test (TWT), the Spastic Paraplegia Rating Scale (SPRS) (11), and the 12-item Multiple Sclerosis Walking Scale (MSWS-12) before and after treatment. Significant post-treatment improvements were recorded in SPRS (*p* = 0.02) and MSWS-12 (*p* = 0.04) scores. After treatment, patients were categorized into two groups: “responders” (*n* = 6 patients), who showed an improvement of >20% in any of the three tests (TWT, SPRS, and MSWS-12), and “non-responders” (*n* = 6 patients), who by definition recorded improvements of < 20% in all tests. However, these results must be interpreted with caution for several reasons: no control group was included and no information was given on the HSP patients' phenotype (pure/complex) or disease duration, and their molecular etiology thus remained uncertain. Overall, the quality of this trial was rated as low (8/22). The second study enrolled five adult patients, all with definite HSP: four with an SPG4 form, and one with an SPG15 form (12). Dalfampridine, 10 mg, was administered orally twice daily for 15 days. Outcome measures were the Modified Ashworth Scale (MAS), 9-hole peg test (NHPT), TWT and the mean time to walk 10 m (10 MWT). All assessments were performed at baseline and at the end of the study. Patients exhibited significantly improved TWT, MAS, and NHPT scores. Like the aforementioned study, this trial was uncontrolled and the sample size small, and overall its quality was considered low (7/22). Interestingly, we have recently studied six patients (three men and three women; median age 23 ± 2 years; mean disease duration 6.5 ± 4 years) harboring biallelic mutations in the SPG11 gene who received 10 mg dalfampridine twice daily for 12 months. Safety profiles were assessed and only minor adverse reactions (fever in one case, gastrointestinal symptoms in two) were observed. We observed a global improvement in motor function as assessed by gait analysis, TWT, and 6-meter walk test (6 MWT), with less fatigue reported on the Modified Fatigue Impact Scale. The patients showed an improved ability to concentrate when performing specific tasks and a subjective improvement in emotional stability, although this was not assessed using standardized clinical tools (FMS, manuscript in preparation). These results are clearly limited by the study design and further controlled studies are necessary to confirm the efficacy of this drug at least in some HSP subtypes.

#### Botulinum Toxin Injection

Intramuscular injection of botulinum toxin type-A (BoNTA) is one of the primary treatments for focal spasticity. In an uncontrolled clinical trial (13), 15 adults with confirmed HSP (8 with SPG4, 1 with SPG3A, 1 with SPG8, and 5 affected by AD-HSP without an identified mutation) and symptomatic calf muscle spasticity were treated with BoNTA injections in each triceps surae (500–750 MU of Dysport®, Ipsen Biopharm Ltd., Wrexham, UK). This was followed by 18 weeks of daily stretching exercises. Before the intervention (T0), and at 4 (T1) and 18 (T2) weeks after it, gait, balance, motor selectivity, and calf muscle tone and strength were tested. The primary outcome measure was comfortable gait velocity, tested barefooted with the 10 MWT. After treatment, mean comfortable gait velocity increased significantly, whereas balance and the other functional measures remained unchanged. Calf muscle tone declined from T0 to T1, an effect that partially persisted at T2, whereas calf muscle strength did not change. The quality of this study was low (6/22). In another study (14) involving 19 unselected adult patients with a probable diagnosis of HSP, treated with BoNTA, and evaluated using the MAS, 17 showed one-point MAS score improvements, whereas a single patient recorded a three-point improvement. In a retrospective study (15), 10 patients with HSP received BoNTA and physical therapy for 5 years. Over time they showed significant improvements both in muscle tone and in baropodometric examination parameters. Overall, the quality of these latter case studies was very low (3/22 and 2/22, respectively).

#### L-Dopa

L-Dopa is an amino acid precursor of dopamine, essentially used in Parkinson's disease. We identified six studies reporting HSP patients who were treated with, and proved partially responsive to, L-Dopa. The first one (16) described two siblings (aged 16 and 18 years) presenting a form of HSP with thin corpus callosum. One patient also had tremor and bradykinesia that were L-Dopa-responsive. The second study (17) described a subject with HSP and parkinsonism harboring biallelic mutations in the SPG11 gene; the patient's parkinsonian features were responsive to 300 mg/day oral L-Dopa. Two further studies (18, 19) described five additional subjects with HSP due to mutations in the same gene in whom treatment with L-Dopa/carbidopa (associated with sapropterin tetrahydrobiopterin in four patients) resulted in a partial improvement of motor symptoms. The final study (20) reported a mother and a daughter with the complicated SPG8 form whose spasticity improved after L-Dopa therapy. The obvious limitation of these reports is that they concerned patients with a limited set of gene mutations (mostly in the SPG11 gene), all studied in open-label, uncontrolled settings. Placebo effects were not taken into account. In addition, information on L-Dopa dosage and outcome measures for assessing efficacy was sketchy and assessments were based only on subjective clinical evaluations. Overall, these studies were considered anecdotal, and of barely acceptable quality (1/22).

#### Cholesterol-Lowering Drugs

Spastic paraplegia type 5 (SPG5) is a rare subtype of HSP caused by recessive mutations in *CYP7B1* encoding oxysterol-7α-hydroxylase, leading to accumulation of neurotoxic oxysterols. We considered a report of two cases plus two RCTs targeting oxysterol accumulation in SPG5. In the first study (21), two adult siblings were treated with cholesterol-lowering agents: one received simvastatin (from 20 to 60 mg/day) for the first 12 months, after which ezetimibe, 10 mg/day, was used in combination with simvastatin, 40 mg/day, for a further year. His sibling, who had not tolerated simvastatin because of side effects, received only ezetimibe, 10 mg/day, for 12 months. In both patients, serum 27-hydroxyoxysterol (27-OHC)—a specific and validated biomarker in SPG5 (1)—significantly decreased, whereas SPRS and muscle strength scores did not change. The quality of this study was rated as very low (2/22). In a more complex study in SPG5 (22), atorvastatin (40 mg/day if patients were aged >18 years, 20 mg/day if < 18 years) was used for 9 weeks in 14 patients and the primary endpoint was a reduction in serum 27-OHC levels. The patients' median age at observation was 40 years, and their median disease duration was 13 years. Atorvastatin, but not placebo, reduced serum 27-OHC by 31.5% (*p* = 0.001). This was deemed to be a high quality (20/22) study, despite being a short-term trial that did not consider any clinical outcome parameters and failed to clarify the long-term impact of cholesterol-lowering agents on disease severity. Finally, a phase II therapeutic study of atorvastatin, 20 mg twice daily, chenodeoxycholic acid (CDCA), 500 mg twice daily, and resveratrol, 40 mg twice daily, was carried out in 12 SPG5 patients (23). It was a three-treatment crossover study with randomization of the six different sequences of the three-treatments. Each patient was studied for a total of 18 months, randomly undergoing the three-treatments each for a period of 2 months, with a washout interval of 4 months. The study included two patients in each arm. The primary outcome measure was reduced plasma 27-OHC levels, whereas the secondary outcome measures were safety profile and changes in oxysterol biomarkers (24S-OHC, 25-OHC). No clinical outcome parameters were considered. The results showed decreased total cholesterol with 40 mg atorvastatin (*p* < 0.001), associated with decreased plasma 27-OHC (*p* < 0.001) and 24S-OHC, but no significant changes in 25-OHC levels. Plasma oxysterols were not significantly altered by CDCA or resveratrol either. However, CDCA led to increased levels of total bile acids (*p* < 0.009), associated with increased serum CDCA, lithocholic acid and ursodeoxycholic acid (*p* < 0.001), and decreased cholic acid and deoxycholic acid (*p* < 0.001). Although well-conducted, the quality of this study was deemed to be moderate (9/22) due to the absence of randomization and blinding. Collectively, these studies offer the first illustrations of a causal treatment strategy in the SPG5 form of HSP. However, aspects still needing to be investigated include the clinical efficacy of cholesterol-lowering drugs and the question of whether biochemical parameters reflect neurological impairment *in vivo*. On the other hand, these preliminary data suggest that it would be useful to evaluate the long-term effects of these drugs in SPG5.

#### Betaine and Folinic Acid

The literature search revealed two case series of patients with complicated HSP caused by methylenetetrahydrofolate reductase (MTHFR) deficiency who showed clinical improvement after treatment with betaine and vitamins. In the first report, Lossos et al. (24) described four patients, from two unrelated families, with severe MTHFR deficiency presenting with complicated HSP. All patients were treated with vitamin B12 (1,000 mg/month), folic acid (15 mg/day), folinic acid (45 mg/day), and betaine anhydrous (6–10 g/day). Betaine treatment resulted in a rapid and sustained reduction in homocysteine levels in all the patients and, over a period of nine to 15 years, improved the conditions of three of them. However, no objective clinical tools were used to assess efficacy. The second report (25) described two MTHFR-deficient adults treated with betaine (9,000 mg/day), folinic acid (45 mg/day), vitamin B12 (1,000 mg/week), vitamin B6 (300 mg/day), and ASA (100 mg/day) as prophylactic agents. One year after starting treatment, their SPRS score decreased from 14 and 15 to 12, respectively. Overall, these clinical studies suggest that betaine and vitamin supplementation may be useful in the rare cases of HSP caused by MTHFR deficiency. However, they were open-label and uncontrolled studies, and their quality was rated as very low (scores of 2/22 and 3/22, respectively).

### Physical Therapy

The effectiveness of physical therapy in patients with HSP was found to be documented only in a small number of case reports and uncontrolled studies.

#### Electrical Stimulation

No RCTs have been conducted to assess the effectiveness of electrical stimulation in HSP treatment. A single published study met the inclusion criteria for this review. The case report in question described a 26-years-old man whose motor function improved after treatment with electrical stimulation (26). Bilateral stimulation of the quadriceps and anterior compartment musculature was performed two to three times per week for 3 months. Gait analysis performed before and after treatment revealed a 27% increase in velocity, associated with increases in cadence, and right step length. The quality of this case report was rated as very low (3/22).

#### Robotic Gait Training

Several robotic locomotion systems are currently available to facilitate gait training in patients with different neurological disorders. We found a single uncontrolled trial whose aim was to test the effectiveness of a robotic-aided intensive training program in adults affected by pure HSP (27). The study included 13 adult patients undergoing rehabilitation treatment (three sessions per week for 6 weeks) using a Lokomat gait orthosis (Hocoma AG, Volketswil, Switzerland). The outcome measures were the following: the Berg Balance Scale (BBS), the Timed Up and Go test (TUG), 6MWT, 10MWT, Physiologic Cost Index (PCI), Hospital Anxiety and Depression Scale, the SF-36 scale, and the MAS. The patients were assessed a week before starting the treatment, at the end of the treatment, and 2 months later, during a follow-up evaluation. At the end of the treatment, statistically significant improvements were observed only on the BBS, 10 MWT, and 6 MWT, whereas the PCI, TUG, and MAS scores did not change. The SF-36 scale showed significant improvements in multiple domains, confirming the positive effects of robotic training on quality of life. The 2-months follow-up scores did not show any change with respect to those recorded at the end of the treatment. In this study there was no comparison with standard therapy, and no switching between treatments; its quality was rated as low (7/22). We also reviewed a case report describing the outcome of robot-assisted gait training combined with physiotherapy in a 28-years-old man with pure HSP (28). This patient underwent 25 training sessions over a period of 6 weeks, after which functional assessments showed some minor improvements in walking speed and balance. The quality of this case report was considered to be very low (2/22). It seems that further studied are needed to establish the role of robot-assisted gait training in the management of HSP.

#### Hydrotherapy

The effectiveness of hydrotherapy treatment in improving locomotor function in patients with late-onset HSP was evaluated in a small, uncontrolled trial (29). Nine HSP patients underwent a 10-week course of hydrotherapy based on 45-min sessions of treatment. Before the treatment, spasticity was measured using the MAS and gait analysis parameters were analyzed in spatiotemporal, kinematic, and kinetic domains. Significant pre- vs. post-therapy differences were observed in spatiotemporal, kinematic, and kinetic measures, and the integration of these measures provides some insight into locomotor inefficiencies and compensatory strategies implemented, during gait, by individuals with HSP. Interestingly, after therapy, a decrease in the rotational range of motion at the hip and knee and an exacerbation of the already abnormal hip internal rotation throughout the gait cycle were observed, suggesting that hydrotherapy might have favored the patients' existing compensatory strategies rather than permitting smoother locomotion. However, the design of this short-term trial was poor and the study was deemed to be of low quality (5/22).

#### Physical Therapy

There exist no clear indications on appropriate types and timing of physical therapy in HSP. One report (30) described two adult siblings with HSP who underwent an intensive 8-weeks stretching, strengthening, and functional exercise program, administered in 60- to 90-min sessions (daily, 6 days/week). The TUG, Functional Reach Test, 10 MWT, and 6 MWT were used as outcome measures, and all showed improvements at the end of the intensive treatment program. Although the results of this study appeared promising, it lacked precise data on the improvements reported. Its quality was rated as very low (2/22).

### Interventional and Surgical Therapies

We did not find high quality studies documenting the effectiveness of interventional and surgical therapies in patients with HSP.

#### Intrathecal Baclofen

Baclofen is a muscle relaxant and antispastic agent that acts by activating GABAB receptors. It can be administered orally or intrathecally. Intrathecal baclofen (ITB) has greater therapeutic efficacy and less systemic toxicity compared with oral preparations. The present literature survey found one open, uncontrolled trial, and two case reports evaluating the efficacy of ITB in HSP patients. In the open study (31), 14 out of 16 adult non-responders to oral antispastic drugs, having responded favorably to an initial intrathecal trial with 50 μg baclofen, were implanted with a pump for intrathecal administration of the drug. The patients were evaluated for lower limb spasticity, walking performance, and complications. The average follow-up period was 25.8 months. The mean baclofen dose was 90 μg/24 h. The treatment was found to reduce lower limb spasticity, measured with the MAS, in all the patients (*p* = 0.000). Walking ability, evaluated using a modified version of the functional walking scale of the Gillette Functional Assessment Questionnaire (*p* = 0.001), was improved. In one of the two case reports (32), MAS score and kinematic and electromyographic analyses showed improvements in an adult HSP patient treated with ITB. In the other case study (33), another patient with HSP reported decreased spasticity, with maintenance of muscle strength, using ITB. Overall, the quality of these studies was rated as low or very low (5/22, 2/22, and 3/22, respectively), showing that there is still a lack of recommendations on the use of ITB in HSP.

#### Dorsal Rhizotomy

Selective dorsal rhizotomy (SDR) is a neurosurgical procedure serving to selectively destroy problematic nerve roots in the spinal cord. It can be used to relieve spasticity in cerebral palsy, but has also been tried in HSP. In an uncontrolled prospective trial, four adults with an undefined form of HSP underwent the procedure (34). Patient evaluation took into account muscle tone and muscle spasms, measured using the MAS, and a spasm frequency scale. Patient data included pre- and post-operative evaluations performed by a multidisciplinary team at 6 months, at 12 months, and then at yearly intervals thereafter. The average lower extremity MAS score decreased after treatment (*p* < 0.01), with no significant difference between the scores at 1 and 2 years after SDR. Lower limb spasm scores also improved significantly. All these improvements remained stable throughout the follow-up period, with no local or general complications appearing in any of the four patients. Although this study provides evidence of the efficacy of SDR, it does not completely answer open questions about the long-term effectiveness and risks of the treatment. Overall, the quality of the study was considered low (6/22). More patients should be followed up for a longer time and larger studies of SDR in specific genetic subtypes should be encouraged. We also reviewed a retrospective study of four HSP children who underwent SDR (35). All the patients showed reduced lower limb spasticity after the procedure, and this change was maintained over long-term follow-up (range 44–252 months). However, progressive functional decline was observed in two siblings diagnosed with the infantile-ascending form associated with mutations in *ALS2*. This study was rated low quality (2/22). On the basis of the above studies, it can be concluded that SDR may possibly be a feasible option for alleviating spinal-related spasticity in uncomplicated HSP, while its effect in complicated HSP seems to be less predictable.

## Discussion

The past decade has seen remarkable advances in the identification of the genes responsible for HSP and in understanding of the molecular pathogenesis of this group of conditions. To date, several pathogenetic mechanisms have been identified (36), including oxidative stress, dysfunction of axonal development, and axonal transport, abnormal lipid metabolism, altered DNA repair, dysmyelination, disrupted autophagy, abnormal cell signaling, and abnormal membrane trafficking. By contrast, curative, to say nothing of preventive, treatments for these disorders continue to be lacking, and there is an urgent need for innovative strategies able to translate growing understanding of the disease mechanisms into improved patient care. Similarly, no recommendations on new therapies are emerging, and it is depressing to note that these patients continue to be treated in the same way as they were in the “pre-genetic era.” In short, symptomatic treatment remains the cornerstone of HSP management. As things currently stand, few trials have evaluated specific HSP treatments, while there is a paucity of robust clinical trial data supporting the efficacy of even the most widely used symptomatic drugs.

The present systematic review, undertaken with the aim of addressing this situation, considered 27 selected studies investigating therapies in HSP: 24 were class IV studies (case reports/series, uncontrolled investigations, or retrospective studies) and three were class III studies (two RCTs and one prospective, placebo-controlled trial with a two-period crossover). Table 1 summarizes them and gives their quality ratings. Only two were considered to be methodologically sound as shown by their high quality scores: one that evaluated the efficacy of gabapentin in HSP, without, however, showing any significant improvement, and another that tested the safety of cholesterol-lowering drugs in SPG5 on the basis of biochemical outcomes, but failed to address short-term motor outcomes. This is too small a number in relation to the constant stream of information emerging at the benchside, not to mention the growing demands of patients, caregivers, and patient associations once a molecular diagnosis has been reached. This is critical in adults suffering a long-lasting disabling disorder but even more serious in children where the paucity of studies and therapeutic attempts reflects a failure to exploit the now more rapid access to molecular diagnoses in young people. Surely, the combined clinical and genetic heterogeneity of the disorder together with inadequacy of natural history studies and validated outcome measures represent a major obstacle to more precise curative studies in HSP. Nonetheless, our review, too, has several limitations. First of all, there actually exist very few RCT studies investigating spasticity treatment, even beyond the hereditary or genetic forms. Second, the patients included in the studies selected for this review are considerably heterogeneous; in some cases, HSP was not even genetically confirmed and the results in a subgroup of HSPs cannot therefore be generalized. Finally, we cannot exclude that our search was incomplete and overlooked significant information coming from smaller studies, case reports, and articles not written in English.

**Table 1:** Overview of the studies included in the literature review.

**Title of paper (Reference)**	**Genetic type of HSP**	**Treatment**	**Type of study**	**Quality score**	**Quality grade**
**PHARMACOLOGICAL TREATMENTS**					
Double-blind crossover trial of gabapentin in SPG4-linked hereditary spastic paraplegia (6)	SPG4	Gabapentin	Prospective double-blind placebo-controlled trial	19/22	Good
The clinical effect of the GABA-agonist, progabide, on spasticity (9)	Not specified	Progabide	Prospective double-blind placebo-controlled trial	16/22	Moderate
Dalfampridine in hereditary spastic paraplegia: a prospective, open study (10)	Not specified	Dalfampridine	Uncontrolled prospective open trial	8/22	Poor
The effects of dalfampridine on hereditary spastic paraparesis (12)	SPG4—SPG15—Not specified	Dalfampridine	Uncontrolled prospective open trial	7/22	Poor
Functional effects of botulinum toxin type-A treatment and subsequent stretching of spastic calf muscles: a study in patients with hereditary spastic paraplegia (13)	SPG4—SPG3—SPG8—Not specified	Botulinum toxin	Uncontrolled prospective trial	6/22	Poor
Botulinum neurotoxin type A injections reduce spasticity in mild to moderate hereditary spastic paraplegia—report of 19 cases (14)	Not specified	Botulinum toxin	Case report	3/22	Poor
Combined treatment Fkt-botulinum toxin type A (Btx-A) in patients with Strumpell-Lorrain disease (15)		Botulinum toxin	Retrospective study	2/22	Poor
Levodopa-responsive parkinsonism in hereditary spastic paraplegia with thin corpus callosum (16)	Not specified	L-Dopa	Case report	1/22	Poor
Novel mutations in SPG11 cause hereditary spastic paraplegia associated with early-onset levodopa-responsive parkinsonism (17)	SPG11	L-Dopa	Case report	1/22	Poor
Neurotransmitter abnormalities and response to supplementation in SPG11 (18)	SPG11	L-Dopa	Case report	1/22	Poor
Exome sequencing expands the mutational spectrum of SPG8 in a family with spasticity responsive to L-Dopa treatment (19)	SPG8	L-Dopa	Case report	1/22	Poor
Dopa-responsive dystonia—clinical and genetic heterogeneity (20)	SPG11	L-Dopa	Case report	1/22	Poor
Treatment of SPG5 with cholesterol-lowering drugs (21)	SPG5	Simvastatin and ezetimibe	Case report	2/22	Poor
Hereditary spastic paraplegia type 5: natural history, biomarkers and a randomized controlled trial (22)	SPG5	Atorvastatin	Randomized controlled trial	20/22	Good
Plasma oxysterols: biomarkers for diagnosis and treatment in spastic paraplegia type 5 (23)	SPG5	Atorvastatin and chenodeoxycholic acid and resveratrol	Phase II therapeutic study	9/22	Moderate
Severe methylenetetrahydrofolate reductase deficiency: clinical clues to a potentially treatable cause of adult-onset hereditary spastic paraplegia (24)	MTHFR^*^	Betaine and folinic acid	Case report	3/22	Poor
Severe 5,10-methylenetetrahydrofolate reductase deficiency: a rare, treatable cause of complicated hereditary spastic paraplegia (25)	MTHFR^*^	Betaine and folinic acid	Case report	2/22	Poor
**PHYSICAL THERAPY**					
Therapeutic electrical stimulation for spasticity: quantitative gait analysis (26)	Not specifid	Electrical stimulation	Case report	3/22	Poor
Robotic gait training improves motor skills and quality of life in hereditary spastic paraplegia (27)		Robotic gait training	Uncontrolled prospective trial	7/22	Poor
Robot-assisted gait training in a patient with hereditary spastic paraplegia (28)		Robotic gait training	Case report	2/22	Poor
The effect of hydrotherapy treatment on gait characteristics of hereditary spastic paraparesis patients (29)		Hydrotherapy	Uncontrolled prospective trial	5/22	Poor
Physical therapy interventions for the patients with hereditary spastic paraparesis. An exploratory case reports (30)		Physiotherapy	Case report	2/22	Poor
**INTERVENTIONAL AND SURGICAL THERAPIES**					
Intrathecal baclofen therapy for the symptomatic treatment of hereditary spastic paraplegia (31)	Not specifid	Intrathecal baclofen	Open uncontrolled study	5/22	Poor
Intrathecal baclofen normalizes motor strategy for squatting in familial spastic paraplegia: a case study (32)		Intrathecal baclofen	Case report	2/22	Poor
Improved gait performance in a patient with hereditary spastic paraplegia after a continuous intrathecal baclofen test infusion and subsequent pump implantation: a case report (33)		Intrathecal baclofen	Case report	3/22	Poor
Improved gait performance in a patient with hereditary spastic paraplegia after a continuous intrathecal baclofen test infusion and subsequent pump implantation: a case report (34)		Dorsal rhizotomy	Uncontrolled prospective trial	6/22	Poor
Selective dorsal rhizotomy for hereditary spastic paraparesis in children (35)		Dorsal rhizotomy	Retrospective study	2/22	Poor

* *MTHFR, methylenetetrahydrofolate reductase deficiency*.

## Future Perspectives

There is no doubt that the search for an effective treatment for HSP, conducted through pathogenetic studies and therapeutic trials, needs to be stepped up. At present, several preclinical avenues of research are offering interesting findings that may open the way for specific pathogenetic therapies in selected forms of HSP. For instance, different studies are evaluating strategies to address spastin deficiency, the cause of SPG4. In an *in vitro* model (37), human-induced pluripotent stem cells derived from fibroblasts of two SGP4 patients were obtained and upregulation of p60 katanin, a microtubule-severing protein, was found to partially compensate for impaired microtubular dynamics seen in SPG4 neurons. In another study, SPG4 homologs in *C. elegans*, fruit fly and zebrafish were studied, and compounds known to modulate endoplasmic reticulum stress (such as phenazine, methylene blue, N-acetyl-cysteine, guanabenz, and salubrinal) partially recovered locomotor defects in mutant models (38). In SPG7 disease, caused by paraplegin deficiency, there has emerged solid proof that intramuscular viral delivery of paraplegin in a knockout mouse model could halt the progression of the neuropathological changes (39). Moreover, in the SPG11 form of HSP, GSK3ß in patient-specific induced pluripotent stem cell-derived cortical neural progenitor cells has been identified as a potential novel target for reversing the disease phenotype (40). A more recent study substantiated the use of Miglustat, which inhibits glycosphingolipid synthesis, in human SPG11 neurons, and a zebrafish knockdown model; the treatment resulted in improved lysosomal clearance and lipid accumulation in neurons and better locomotion in treated larvae (41). These promising data are begging to be used to improve clinical efficacy. It is equally important to invest research money on multicenter natural history studies and to validate physical outcome measures and biomarkers for the different forms. Tests, such as 6 MWT, TUG, or 10 MWT as well as specific scoring systems or scales adopted to measure fatigue and quality of life might serve in general in HSP but could be inappropriate in more complex setting or in young children. Today, with pathogenetic research advancing all the time and personalized gene therapy approaches just around the corner, well-designed clinical trials of symptomatic drugs for HSP treatment are clearly lagging behind. There is a need for RCTs with sufficiently large samples and precise biological outcome measures, to evaluate the efficacy of the treatments already in use in current neurological practice.

## Author Contributions

MB, GD, and AI drafted the manuscript, performed literature search, assessed scores, and critically revised results. AF and FS revised the work strategy and score assessments, and edited the manuscript. FS conceived the study, oversaw data acquisition, supervised the initial draft, and critically revised the manuscript.

### Conflict of Interest Statement

The authors declare that the research was conducted in the absence of any commercial or financial relationships that could be construed as a potential conflict of interest.
