# Supplementary material for: Management of Hereditary Spastic Paraplegia: A Systematic Review of the Literature
Source: Front Neurol. 2019 Jan 22;10:3. doi: 10.3389/fneur.2019.00003 (PMC6349696; doi:10.3389/fneur.2019.00003)
Supplement: Supplementary file 1 [file Data_Sheet_1.doc]

**Appendix. Criteria used for assessment of methodological quality of included studies***

**A**: Treatment adequately concealed prior to allocation

**B**: Participants who withdrew or were excluded after allocation were described and included in an 'intention to treat' analysis

**C**: Assessors were blind to assignment status

**D**: Treatment and control groups were comparable at entry

**E**: Participants were blind to assignment status following allocation

**F**: Treatment providers were blind to assignment status?

**G**: Care programs, other than the trial options, were identical

**H**: Inclusion and exclusion criteria for entry were clearly defined

**I**: Interventions were clearly defined

**J**: Outcome measures were clearly defined

**K**: Diagnostic tests used in outcome assessments were clinically useful

* All items are scored 0 to 2 and are taken with modifications from Rietberg MB, Brooks D, Uitdehaag BM et al. (Ref. 4) (see supplementary appendix 2)
